# Direct Measurements of Overlooked Long-Range Interactions near Zwitterionic and Nonionic Polymer Brushes

**DOI:** 10.1021/acsmacrolett.5c00043

**Published:** 2025-03-31

**Authors:** Jiahao Wu, Feng Cao, Manjia Li, Wei Liu, Kohji Ohno, To Ngai

**Affiliations:** † Department of Chemistry, The Chinese University of Hong Kong, Shatin, N.T., Hong Kong 999077, China; ‡ Department of Chemical and Biological Engineering, The Hong Kong University of Science and Technology, Clear Water Bay, Kowloon, Hong Kong 999077, China; § The Key Laboratory of Synthetic and Biological Colloids, Ministry of Education & School of Chemical and Material Engineering, Jiangnan University, Wuxi 214122, China; ∥ Department of Materials Science, Graduate School of Engineering, Osaka Metropolitan University, Sakai, Osaka 599-8531, Japan

## Abstract

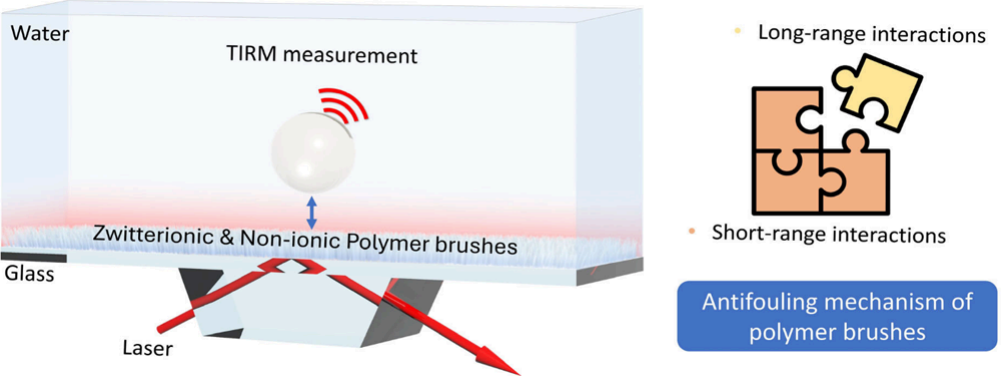

Current research on the antifouling mechanisms of “electrically neutral” polymer brushes predominantly emphasizes thermodynamically unfavorable short-range interactions. However, our study reveals the critical importance of long-range interactions. By utilizing zwitterionic poly(carboxybetaine methacrylate) (PCBMA) and nonionic poly[oligo(ethylene glycol) methyl ether methacrylate] (POEGMA) brushes as model systems, we employed total internal reflection microscopy (TIRM) to directly measure interactions with contaminants. Surprisingly, even seemingly neutral polymers exhibit significant electrostatic interactions with nearby contaminants—a fact that has been largely overlooked in this field. Our findings challenge the prevailing assumption of charge absence on surfaces grafted with antifouling polymer brushes and investigate how external stimuli (such as ionic strength and polymer conformation) affect these long-range interactions. In conclusion, this study presents a novel approach to exploring long-range interactions near polymer-grafted surfaces, offering valuable insights for the development of antifouling materials and biomedical applications in the future.

Approaches that impart antifouling properties to surfaces have garnered great traction with researchers over the past few decades. Among them, due to its low cost, excellent performance, and high versatility, grafting antifouling polymer brushes on surfaces has become one of the most popular approaches.^1^ For instance, the use of polymer brushes on biomedical implants can effectively reduce bacterial colonization, leading to milder infections and lower antibiotic resistance.^2^ In marine industries, similarly, coating hulls with polymer brushes can mitigate the accumulation of aquatic organisms, resulting in fuel and cost savings.^3^ In the evolving research landscape, a wide range of antifouling polymer brushes has emerged, with two primary categories garnering substantial attention and practical applications. The first category includes polyethylene glycol (PEG), known as the gold standard, and its derivatives.^4^ These nonionic antifouling polymers share common characteristics outlined by Whitesides and co-workers, including hydrophilicity, hydrogen bond formability, and electrical neutrality. The second category comprises zwitterionic polymers like polybetaines and polyampholytes.^5^ These rising stars are also considered to exhibit similar attributes that follow the Whitesides’ rules, thus making them effective antifouling materials.^6^

As for the underlying mechanism, our current understanding is based on the characteristics of polymer brushes mentioned above, which mainly focuses on thermodynamically unfavorable interactions between polymer brushes and contaminants. First, as contaminants approach, they compress the polymer brushes and restrict their free mobility. This entropically unfavorable process leads to steric repulsion from the polymer brushes.^7^ Additionally, a dense hydration layer can be formed around the polymer brushes. The dehydration of this layer is also thermodynamically unfavorable, and thus, the adhered water molecules can act as a barrier to prevent contaminants from fouling.^8^ Notably, the mechanism described above occurs over a very short-range, typically less than 1 nm.^9^ However, in areas like colloid science, the impact of long-range interactions on particle repulsion and system stability cannot be overlooked. Hence, the intriguing question arises regarding the specific role of these forces in the antifouling mechanism of polymer brushes.

Surprisingly, prior investigations have inadequately addressed this aspect, possibly due to the presumption of electrical neutrality. Although some hints have shown that surfaces grafted with antifouling polymer brushes might not be entirely devoid of charge, like the negative ζ-potential of polymer-modified surfaces^10^ or the easier adsorption of positively charged contaminants to PEG-derived brushes,^11^ the discovery of surface electronegativity is often attributed to incomplete charge shielding. Recent studies have raised questions about the charge-carrying properties of these “natural” polymers themselves, with findings indicating consistent negative ζ-potentials of zwitterionic sulfobetaine-modified silica nanoparticles.^12^ Researchers proposed that the negative charge comes from the stabilization of siloxide from residual surface silanols by the quaternary amine in the sulfobetaine structure.^12^ However, further analysis is challenging, as even the resolution of atomic force microscopy (AFM) is still difficult to obtain the force-versus-distance curves near these “neutral” surfaces to reliably confirm the roles of electrostatic interaction that vary with ionic strength, which has led to a long-standing neglect of in-depth studies in this area.^13^

Therefore, it is essential to develop a method for quantitatively evaluating the long-range antifouling mechanism before short-range interactions come into play. We utilized a highly sensitive technique called total internal reflection microscopy (TIRM) to directly characterize long-range interactions near zwitterionic poly(carboxybetaine methacrylate) (PCBMA) and nonionic poly[oligo(ethylene glycol) methyl ether methacrylate] (POEGMA) brushes grafted on glass slides (Figure 1a).^14−17^ In TIRM measurements, the potential-versus-distance curves are measured based on the probability of tracer position near the polymer brushes. Instead of monitoring the mechanical response of a cantilever, measuring near-surface interactions statistically greatly improves our resolution of near-surface interactions to the order of *k*_B_*T*, which allow us to clearly unmask the underestimated role of long-range interactions near polymer brushes (Figure 1b).^18−22^ As illustrated in the SI section, continuous monitoring of particle motion near the substrate is the key to TIRM measurements. Probes used in this work are sulfated polystyrene microspheres, which are commonly used in many previous works. These microspheres possess sufficient surface charge and share the same type of charge as the substrates. Additionally, they have suitable density, diffusion range, and refractive index. This combination enables clear and continuous monitoring of the scattering signals from the freely moving microspheres near the substrate, even when the substrate’s surface charge is not substantial. Our findings novelly reveals the presence of electrostatic interactions near these seemingly ″neutral″ polymer brushes, significantly influencing contaminant distribution near the antifouling surfaces. Through our investigation into the origins of charge, we proposed that the polymer brushes themselves carry charge and affect their interactions with contaminants. Additionally, we delved into other factors such as van der Waals attraction and polymer conformation. Overall, this study introduces a reliable approach for analyzing long-range interactions near antifouling polymer brushes, providing a deeper understanding of their mechanism and offering valuable insights for shaping the future design of antifouling polymer brushes across diverse research domains.

**Figure 1:**
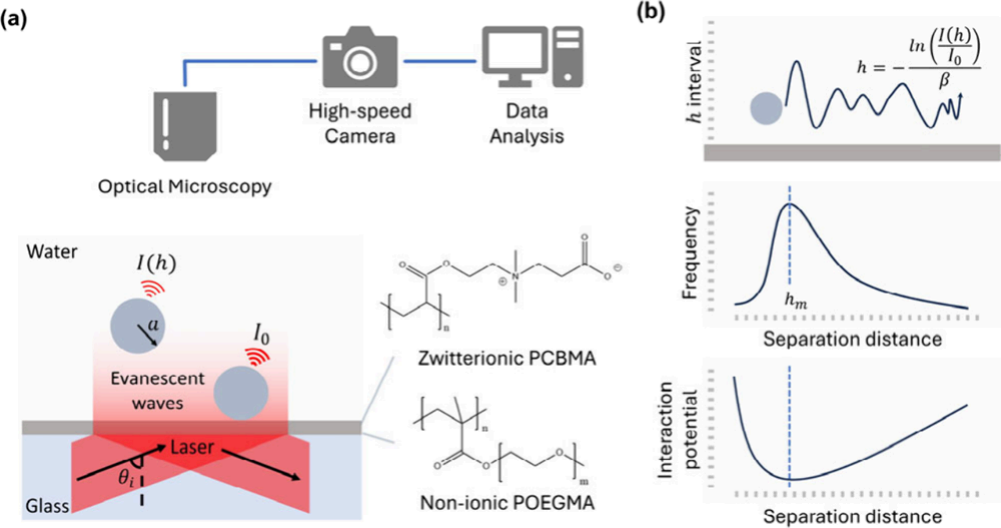
Schematic illustration of using total internal reflection microscopy (TIRM) to measure long-range interactions near zwitterionic PCBMA and nonionic POEGMA polymer brushes in aqueous solution: (a) Illustration of experimental setup. (b) Principle of data analysis. The scale in the illustration is not representative of the actual size.

We first focus on the long-range interactions near surface-grafted zwitterionic PCBMA brushes. The brushes were grafted onto glass slides via atom transfer radical polymerization (ATRP), with *M*_n_ = 52000 g/mol and a graft density of 0.2 chains/nm^2^. Figure 2a illustrates the *k*_B_*T*-level interaction potential between the PS microspheres and the PCBMA brushes across various salt concentrations. Despite appearing weak, *k*_B_*T*-level interactions are significant enough to influence the diffusion of common contaminants such as proteins, bacteria, and microalgae near the polymer brushes. The interaction potential curves in Figure 2a can be divided into two segments based on their slope, indicating repulsion and attraction between the microspheres and the polymer brushes. The point *h*_m_, where the slope is zero, represents the equilibrium between the repulsion and the attraction. At a low salt concentration (0.1 mM), the exceptional sensitivity of TIRM allowed us to detect a strong repulsion when the microspheres were more than 300 nm from the polymer brushes. Due to the strong dipole–dipole interaction within the polymer chains, PCBMA brushes are unswollen under low ionic strength conditions.^15,17^ Our reported characterization indicates that the thickness of the PCBMA brushes here, both in dry conditions and when immersed in a 0.1 mM NaCl solution, is less than 20 nm. Hence, the scenario in which the collapsed PCBMA brushes could swell significantly enough to induce direct short-range repulsions, such as steric repulsion, seems improbable. The antifouling mechanism of PCBMA brushes under low ionic strength conditions is likely associated with some kinds of long-range interaction.

**Figure 2:**
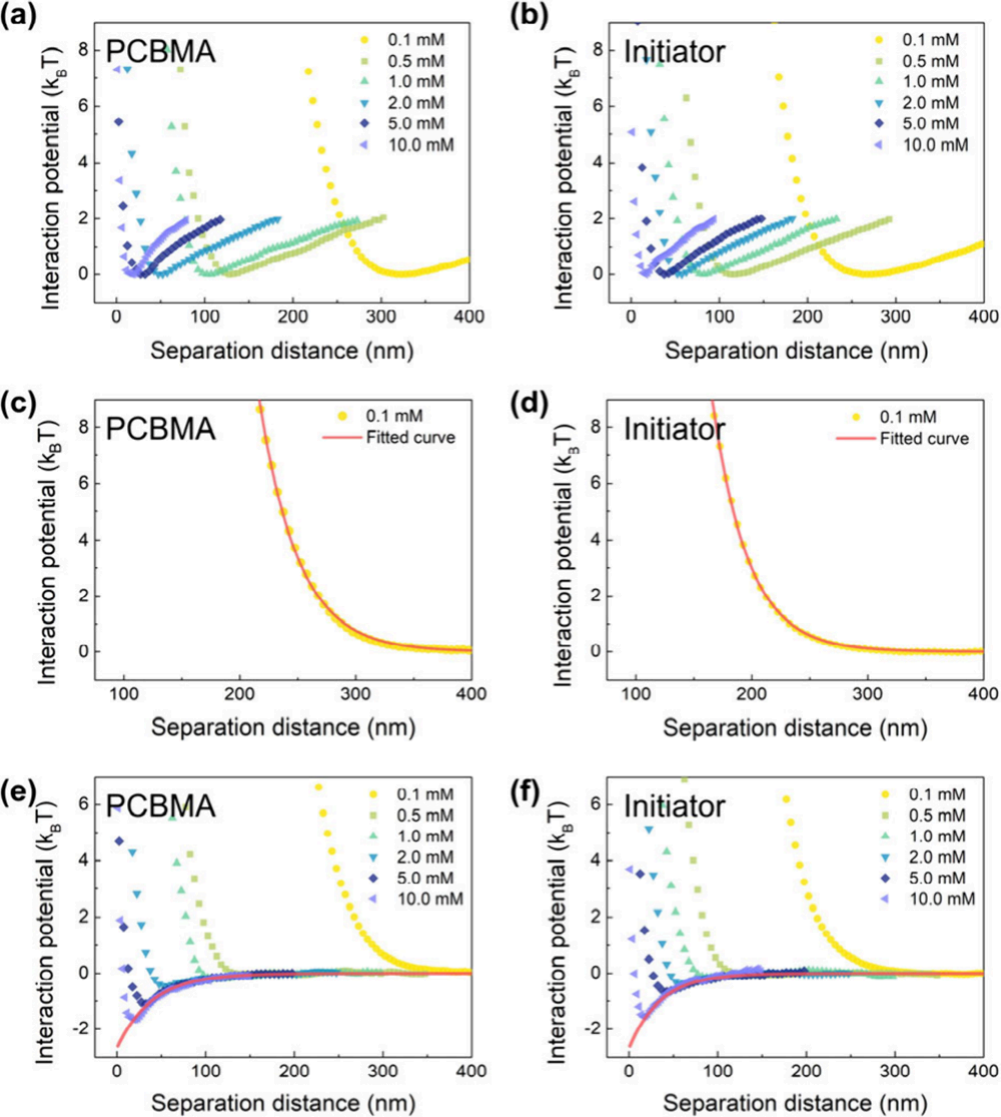
(a, b) Interaction potential between the microspheres and the PCBMA/initiator-grafted surface in NaCl solution at varying concentrations, respectively. (c, d) Interaction potential between the microspheres and the surface in a 0.1 mM NaCl solution, derived from (a) and (b), respectively, after deducting the net gravity of the microspheres. The solid lines represent the fitting of the experimental data using eq S6. (e, f) Interaction potential between the microspheres and the PCBMA/initiator-grafted surface in NaCl solution at varying concentrations with the net gravity of the microspheres deducted. The solid lines in (e) and (f) are obtained by fitting the attraction part exponentially. The fitted results are shown in eqs 1 and 2.

As ionic strength further increases, the interaction potential curves reveal a steady decrease in *h*_m_ (Figure 2a and Table 1). While the gravitational attraction remains constant during the measurements, the decrease in *h*_m_ indicates a weakening of the repulsion between the polymer brushes and microspheres. A plausible explanation for this salt-responsive long-range interaction is electrostatic interaction. Then, by fitting the repulsive parts of the interaction curves with the appropriate eq (eq S6), we obtained corresponding Debye length κ^–1^ in each group, which also decreases gradually with increasing salt concentration (Table 1). This observation is also indicative of the presence of electrostatic repulsion between the polymer brushes and the microspheres. While the PS microspheres carry negative charge because of their interfacial sulfate groups, the PCBMA-grafted surface is supposed to be negatively charged accordingly. After the ionic strength exceeded 10 mM, all particles settled on the polymer brushes, because the gravitational attraction and the van der Waals attraction could not be balanced by the electrostatic repulsion any longer. The settlement process observed in our system suggests that the conventional antifouling mechanisms involving steric repulsion and hydration barriers were ineffective in preventing it. This indicates a shift in the dominant antifouling mechanism in our system, with long-range electrostatic repulsion superseding the short-range interactions described in previous theories. Consequently, when studying the antifouling processes of contaminants such as microplastics, which share similarities with the PS microspheres, relying solely on traditional mechanisms could obscure the substantial impact of long-range interactions on their location distribution near the antifouling surfaces.

**Table 1:** *h*_m_ Values and Fitted Debye Lengths κ^–1^ for the PCBMA/Initiator-Grafted Glass Slide

substrate	NaCl concn (mM)	*h*_m_ (nm)	fitted κ^–1^ (nm)	theoretical κ^–1^ (nm)
PCBMA-grafted surface	0.1	322.5	31.1	30.4
	0.5	127.5	14.3	13.6
	1.0	102.5	9.3	9.6
	2.0	52.5	8.8	6.8
	5.0	32.5	7.0	4.3
	10.0	18.75	3.4	3.0
initiator-grafted surface	0.1	267.5	30.4	30.4
	0.5	117.5	13.9	13.6
	1.0	82.5	11.2	9.6
	2.0	57.5	8.5	6.8
	5.0	37.5	6.0	4.3
	10.0	16.25	3.0	3.0

Nonetheless, there remained a question: from which part of the PCBMA-grafted surface does the electrostatic repulsion originate—the polymer brushes or the underlying glass slide? To address this query, we delved further into the interactions between the PS microspheres and an initiator-grafted glass slide. As illustrated in Figure 2b, we observed a notable presence of electrostatic repulsion, attributed to the negative charge carried by the initiator-grafted glass slide in the NaCl solution due to residual -SiO- groups on the surface. The electrostatic repulsion at an ionic strength of 0.1 mM was fitted using eq S6 and simultaneously compared with the electrostatic repulsion observed near the PCBMA-grafted surface. The electrostatic repulsion investigated here is far away from the surfaces so that it is unaffected by near-wall attractions, ensuring the reliability of the fitting process. According to eq S7, a larger fitted B value in eq S6 corresponds to a larger absolute surface potential. Through the comparison of fitted B values, we find that the surface potential strengthens after being grafted with PCBMA, as evidenced by the fitted B value of 5605 *k*_B_*T* for the PCBMA-grafted surface (Figure 2c), while the value for the initiator-grafted surface stood at only 1915 *k*_B_*T* (Figure 2d).

In TIRM measurements, as shown in Figure 1a, the location of the stuck PS micropheres is the reference for separation distance in following data analysis. For PCBMA brushes, they are collapsed under our testing conditions and cannot be further compressed by the stuck PS microspheres. Consequently, the fitting of the B value can accurately characterize the properties at the outer edge of both surfaces and clarifies the previous question: the main source of the negative charge on the PCBMA-grafted surface should be the polymer brushes themselves. In the case where the long-range repulsion is provided by the glass surface and the zwitterionic PCBMA layer undergoes strong screening, the fitted B value of the interaction between tracers and PCBMA will have a smaller value than 1915 *k*_B_*T*. Regarding the underlying reasons, first, the isoelectric point of PCBMA is reported to be 6.4, slightly lower than 7.^23^ This suggests that the dominant deprotonation of the carboxyl group imparts an overall negative charge to the polymer brushes in NaCl solutions. Another explanation, proposed by Mondo et al., suggests that the negative charge is due to the stabilization of siloxide from residual surface silanols by the quaternary amine in the sulfobetaine structure.^12^ Besides, preferred ionic adsorption on zwitterionic polymer brushes has been reported as a potential reason for the negative zeta potential of these “neutral” polymers.^24,25^ To summarize, the experimental results in this part validate the negative charge on the PCBMA brushes and highlight the significance of electrostatic repulsion in its antifouling mechanism. This serves as a reminder that researchers might need to carefully consider the assumption of polymer electroneutrality when studying the antifouling properties of zwitterionic polymers in the future.

In addition to electrostatic repulsion, we have identified the involvement of other types of interactions in influencing the interaction potential between the PS microspheres and the antifouling surface. In Figure 2a, it is evident that the slope of the attractive region increases progressively as the microspheres approach the PCBMA brushes. A similar trend is observed when measuring interactions near the initiator-grafted surface. Since the net gravity acting on the PS microspheres remains relatively constant, we attribute this variation to the additional retarded van der Waals attraction between the microspheres and the bottom surface. Similar to other researchers, we employ an empirical exponential function to achieve the fitting for the attraction.^20,22^ The solid red curves in Figure 2e–f demonstrate the successful description of the van der Waals attraction between the microspheres and the PCBMA/initiator-grafted surface using eqs 1 and 2, respectively.
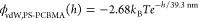
1
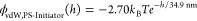
2

The pre-exponential factors in these two exponential equations are around −2.7*k*_B_*T* in our cases, which is close to the value of −3*k*_B_*T* reported by Bevan and Prieve when they measured PS-glass and PS–PS interactions.^20^ This similarity suggests that the van der Waals attraction between the microspheres and the PCBMA/initiator-grafted surface follows a similar trend. Regarding the decay length, it increases slightly from 34.9 to 39.3 nm after grafting the polymer brushes, which is similar to previous findings when a glass slide was spin-coated with a polymer layer.^20^ These results indicate that the PCBMA brushes do have an effect on the van der Waals attraction, but the collapsed conformation of the brushes makes their impact closer to that of a dense polymer coating. In principle, if the polymer brushes are fully swollen, the van der Waals attraction will be weakened more significantly, thereby enhancing the overall antifouling performance of the polymer brushes. Additionally, dipole–dipole interactions, a component of van der Waals attraction, may also play an important role to this additional attraction. According to a recent study by Sarker et al., the spacing between the opposite charged moieties results in dipole moment of a zwitterion.^26^ When the zwitterionic spacing of the polymer brushes is shorter or longer, the dipole moment of zwitterionic materials, as well as their dipole–dipole interactions with the foulants, becomes weaker or stronger accordingly.^26^

Similar to PCBMA brushes, POEGMA brushes were grafted onto the glass slides via ATRP, with *M*_n_ = 133000 g/mol and a graft density of 0.1 chains/nm^2^. While PEG-derived polymer brushes are typically perceived as neutral, as demonstrated in the prior section, we have also observed a notable electrostatic repulsion between the PS microspheres and the nonionic POEGMA brushes. This repulsion effectively prevents fouling of PS microspheres on the surface, suggesting that the POEGMA brushes are negatively charged in NaCl solution. However, the underlying reason for this phenomenon remains unclear.^27,28^ One possibility is that preferred ion adsorption on the POEGMA brushes contributes to the negative charge, similar to what has been observed with PCBMA brushes.^24^ In a study by Wu et al., the negative charge of POEGMA-grafted surfaces was utilized to develop membranes for demulsification and fouling resistance during emulsion separation.^27^

Once again, the fitted B value of the interaction curve measured in 0.1 mM NaCl solution (Figure 3b) is utilized to investigate the source of the surface charge. Based on our previous characterization, it is known that the POEGMA brushes are swollen in NaCl solution with low ionic strength.^17^ As swollen POEGMA brushes can be compressed by stuck PS microspheres, the separation distance we obtained in TIRM measurements, named *h*_optical_, is larger than the separation distance between free-moving microspheres and the hydrodynamic boundary of the swollen polymer brushes, denoted as *h*_hydro_. The comparison between these two axes of separation distance is depicted in Figure 3c. Then, as illustrated by the navy plots in Figure 3b, an offset of 83 nm, as determined in our previous study, is added to the curve to characterize the charge distribution at the outer hydrodynamic edge of the brushes.^17^ The fitted B value of the PS–POEGMA interaction, when considering the hydrodynamic separation distance *h*_hydro_, is 648 *k*_B_*T*, which is substantially smaller than the B value of the PS–PCBMA interaction, indicating that the surface potential generated by nonionic polymer brushes is significantly smaller than the potential generated by zwitterionic polymer brushes.

**Figure 3:**
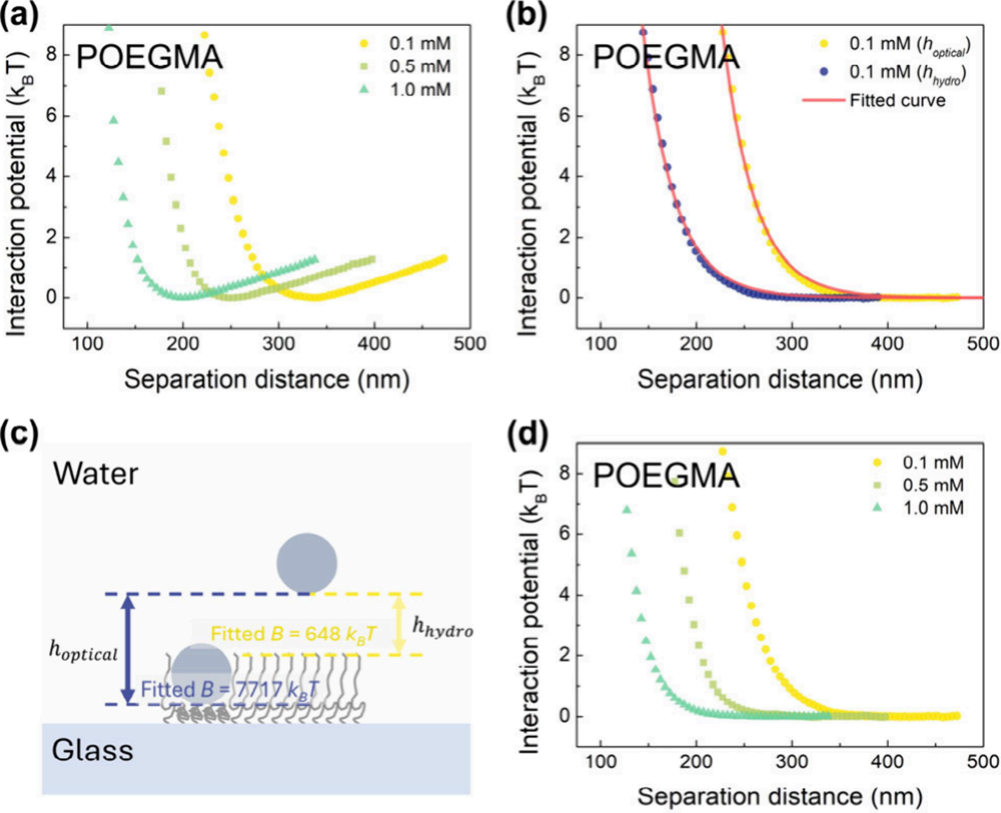
(a) Interaction potential between PS microspheres and the POEGMA-grafted surface at different NaCl concentrations. (b) Interaction potential between PS microspheres and the POEGMA-grafted surface in 0.1 mM NaCl solution with net gravity of the tracers deducted. Solid lines are fits to the experimental data according to eq S6. (c) Schematic illustration comparing fitting data in (b) with different localization approaches. The scale in the illustration is not representative of actual size. (d) Interaction potential between PS microspheres and the POEGMA-grafted surface at different ionic strengths with net gravity of the tracers deducted. No additional van der Waals attraction was detected in the measurements.

The possibility that the negative charge comes from the substrate is small. First, the stretched POEGMA chains in the 0.1 mM NaCl solution create a thick polymer brush layer that effectively screens the negative charge on the substrate. Second, if we consider the optical separation distance, the fitted B value would increase to 7117*k*_B_*T*. This indicates that even if we equate the charged plane to the edge of the compressed POEGMA (Figure 3c), the required surface potential to repel the tracers would exceed the surface potential of the substrate with immobilized initiators. Therefore, similar to the previous findings with PCBMA brushes, it is more plausible that the negative charge originated from the POEGMA brushes rather than the substrate. These results highlight that the long-range electrostatic interaction near nonionic PEG-derived polymer brush surfaces can have a more significant impact than initially anticipated.

Furthermore, as shown in Table 2, the fitted values of the Debye length are larger than the theoretically predicted values, especially at higher ionic strengths. For example, when the salt concentration is 1.0 mM, the theoretical value of Debye length, κ^–1^, is only 9.6 nm, while the experimental value reaches up to 19.6 nm, which is more than double the theoretical value. we employed a biexponential equation to fit the curves and investigate whether the significant increase was due to the retarded van der Waals force mentioned earlier. However, we do not observe any additional exponential attraction that could account for the change. This suggests that the role of the retarded van der Waals attraction is negligible in this context. Therefore, the deviation in the Debye length is more likely attributable to factors such as the charge distribution inside the swollen POEGMA brushes and the diffusion layer outside the brushes.^29^ Murakami et al. have found a similar increase in Debye length when measuring the interaction between particles coated with polyelectrolyte brushes.^29^ Our findings in this study reveal that this deviation also occurs when the polyelectrolyte brushes are replaced by nonionic polymer brushes. From the perspective of antifouling properties, the larger apparent Debye length of polymer-grafted surfaces leads to a slower decay of electrostatic repulsion.

**Table 2:** *h*_m_ Values and Fitted Debye Lengths κ^–1^ for the POEGMA-Grafted Glass Slide

substrate	NaCl concn (mM)	*h*_m_ (nm)	fitted κ^–1^ (nm)	theoretical κ^–1^ (nm)
POEGMA-grafted surface	0.1	337.5	34.7	30.4
	0.5	252.5	20.8	13.6
	1.0	197.5	19.6	9.6

Compared to the collapsed PCBMA brushes, we observed another intriguing phenomenon: the PS microspheres near the swollen POEGMA brushes tend to lose their free fluctuation more easily once the ionic strength exceeds 1.0 mM. This can be attributed to the weaker electrostatic repulsion and stronger bridging attraction between the polymer chains and the rigid surface of the PS microspheres in the case of POEGMA brushes, even though the van der Waals attraction in this system is weak. While swollen polymer brushes are typically associated with providing effective steric repulsion and enhancing the surface’s resistance to fouling, our findings suggest that the antifouling mechanism of polymer brushes is multifaceted. For instance, in scenarios involving contaminants like rigid microplastics, the swollen conformation of polymer brushes might reduce the surface’s antifouling properties due to the additional bridging attraction. From our point of view, antifouling performance appears to be determined by the interplay of various factors, encompassing not only short-range interactions but also long-range interactions, polymer conformation, surface properties, external conditions, and more.

To sum up, in this study, we investigate the long-range nonspecific interactions near surfaces grafted with two distinct types of polymer brushes (zwitterionic PCBMA and nonionic POEGMA) at different ionic strengths using total internal reflection microscopy. The findings based on our innovative approach are very good complements to the canonical antifouling mechanism based on short-range interactions.

First of all, contrary to the conventional assumption of electrical neutrality in polymer brushes, our research reveals that both zwitterionic and nonionic polymer brushes exhibit significant long-range electrostatic interactions, impacting the distribution of PS microspheres near the surfaces and influencing the overall antifouling process in our experimental setups. By analyzing the fitted B values of various potential curves of electrostatic interactions, we determine that the negative charge of the antifouling surfaces likely originates from the polymer brushes themselves rather than the substrate. This suggests that the assumption of electrical neutrality in polymer brushes might need more careful reconsideration in future studies. We also observe that the conformation of the polymer brushes and external stimuli, such as ionic strength, can alter the significance of certain types of interactions or the decay behavior of long-range interactions near the surface.

Additionally, our findings may be applicable to a broader range of antifouling polymers. For instance, polyampholytes, which are pseudozwitterionic polymers, struggle to achieve a precise balance of positive and negative charges.^30^ This could result in more pronounced long-range electrostatic interactions between polyampholytes and foulants compared to the cases discussed in this work. In contrast, poly(ylides), a new class of zwitterionic antifouling polymers, feature shorter charge separations in their chemical structures than PCBMA.^30^ This leads to decreased electrostatic potential and dipole moments,^26^ potentially weakening both van der Waals and long-range electrostatic interactions near the polymers. Therefore, it is crucial for future research to include more polymers, parameters, and methods to achieve a more systematic understanding of long-range interactions near antifouling polymer brushes.

In conclusion, our research does not diminish the significance of short-range interactions in the antifouling mechanism of polymer brushes. Rather, we highlight that the overall antifouling performance is shaped by a complex interplay of factors, including short-range and long-range interactions, polymer conformation, pollutant types, external conditions, and more. Further studies are essential to deepen our understanding of antifouling systems in this field. Beyond antifouling applications, the surface charge of polymer-modified surfaces plays crucial roles in various fields, including drug delivery systems where efficient cellular uptake is crucial. Therefore, we anticipate that our findings will offer valuable insights for the design and utilization of polymer brushes across diverse research domains.

## Supporting Information Available

General information on materials, synthesis of polymer brushes, principle and setup of total internal reflection microscopy (PDF)
