# Supplementary material for: Direct Measurements of Overlooked Long-Range Interactions near Zwitterionic and Nonionic Polymer Brushes
Source: ACS Macro Lett. 2025 Mar 31;14(4):502–8. doi: 10.1021/acsmacrolett.5c00043 (PMC12004931; doi:10.1021/acsmacrolett.5c00043)
Supplement: Supplementary file 1 — mz5c00043_si_001.pdf [file mz5c00043_si_001.pdf]

Supporting Information

Direct measurements of overlooked long-range interactions near zwitterionic and non-ionic polymer brushes

Jiahao Wu[a], Feng Cao[a], Manjia Li[b], Wei Liu[c,]* Kohji Ohno[d], and To Ngai[a,]*

[a]Department of Chemistry, The Chinese University of Hong Kong, Shatin, N.T., Hong Kong, China
[b]Department of Chemical and Biological Engineering, The Hong Kong University of Science and Technology, Clear Water Bay, Kowloon, Hong Kong, China
[c]The Key Laboratory of Synthetic and Biological Colloids, Ministry of Education & School of Chemical and Material Engineering, Jiangnan University, Wuxi 214122, China
[d]Department of Materials Science, Graduate School of Engineering, Osaka Metropolitan University, Sakai, Osaka 599-8531, Japan

Corresponding author
*Prof. Wei Liu, email: weiliu@jiangnan.edu.cn
*Prof. To Ngai, email: tongai@cuhk.edu.hk

Materials

Sulfate polystyrene (PS) microspheres (particle diameter: 5.6 μm, ζ-potential: -100 mV, Invitrogen, USA), ethanol (Duksan, South Korea), sodium chloride (Scharlau, Spain)hydrogen peroxide (30% v/v, Scharlau, Spain), sulfuric acid (98% v/v, RCI Labscan, Thailand), and deionized water (18.2 MΩ·cm, Millipore, USA) were utilized as received without further purification. Glass slides (BK-7, 75 mm × 25 mm, premium-grade, Fisher Scientific, USA) were cut into 25 mm × 25 mm pieces and underwent initial cleaning by immersion in Piranha solution (H_2SO_4:30% H_2O_2 = 3:1) at room temperature for 2 hours. Following thorough rinsing with water and ethanol, the slides were dried using nitrogen gas and subsequently cleaned for 5 minutes in an ultraviolet-ozone plasma cleaner (PDC-002, Harrick Plasma, USA) before use. Surface-initiated atom transfer radical polymerization (SI-ATRP) was applied to graft the PCBMA and POEGMA brushes onto the glass slides. Detailed procedure and characterization can be found in our previous publications.[1-3]

Synthesis of PCBMA brushes

The synthesis protocol of PCBMA brushes follows the methodology described in our earlier research.[3] Firstly, carboxybetaine methacrylate (CBMA, 6.4 g) and ethyl 2-bromoisobutyrate (EBiB, ATRP free initiator, 5.4 mg) were dissolved in 2,2,2-trifluoroethanol (14 mL) and subjected to degassing with argon. This mixture was introduced into the reaction vessel containing 2,2′-bipyridyl (Bpy, 18 mg), CuBr (8 mg), and the BPE-modified glass substrate under an argon atmosphere. The ATRP reaction proceeded for 24 hours at 60 °C. Post-reaction, the PCBMA brush-modified substrate underwent a series of washes with methanol, ultrasonication in methanol, multiple rinses with methanol, water, and acetone, before being dried using a nitrogen gas flush. The number-averaged molecular weight (M_n), weight-averaged molecular weight (M_w), and molecular weight distribution (M_w/M_n) of the resultant free PCBMA from EBiB were determined using gel permeation chromatography (GPC, GPC-101, Shodex, Tokyo, Japan) equipped with two columns (GPC SB-804, Shodex) and a differential refractometer (RI-101, Shodex). The GPC system employed a mixed solution of water/acetonitrile (3/2 ratio) containing 10 mM LiCl as the eluent, running at a flow rate of 0.8 mL/min. The calibration of the GPC system utilized poly(ethylene glycol) (PEG) standards. Finally, the PCBMA brushes (with M_n = 52,000 g/mol and graft density of 0.2 chains/nm^2) were successfully grafted onto the glass slides. The dry thickness of the POEGMA brushes was determined to be 13 nm through atomic force microscopy (XE-100, Park Systems, South Korea) in our previous study.[3] The thickness of these brushes in 0.1 mM, 1.0 mM, and 10 mM NaCl solutions has also been characterized using another atomic force microscopy (BioScope Resolve, Bruker) and measured at 15.8 nm, 16.4 nm, and 17.1 nm, respectively.[3]

Synthesis of POEGMA brushes

The synthesis protocol of POEGMA brushes also follows the methodology described in our earlier research.[3] Firstly, oligo(ethylene glycol) methyl ether methacrylate (OEGMA, M_n = 500,

9.8 g) and EBiB (0.4 mg) were dissolved in anisole (10 mL) and subjected to degassing with argon. This solution was introduced into the reaction vessel containing 4,4'-dinonyl-2,2'-bipyridine (dNbipy, 94 mg), CuCl (43 mg), and the BPE-modified glass substrate under an argon atmosphere. The ATRP reaction was conducted for 20 hours at 60 °C. Post-reaction, the POEGMA brush-modified substrate underwent a series of washes with methanol, ultrasonication in methanol, multiple rinses with methanol, water, and acetone, before being dried using a nitrogen gas flush. The M_n, M_w, and M_w/M_n values of the resulting free POEGMA were determined through gel permeation chromatography (GPC, GPC-101, Shodex, Tokyo, Japan) equipped with two columns (GPC KF-806L, Shodex) and a differential refractometer (RI-101, Shodex). N,N-Dimethylformamide (DMF) served as the eluent at a flow rate of 0.8 mL/min, with calibration of the GPC system performed using poly(methyl methacrylate) (PMMA) standards. Finally, the POEGMA brushes (with M_n = 133,000 g/mol and graft density: 0.1 chains/nm^2) were successfully grafted onto the glass slides. The dry thickness of the POEGMA brushes was determined to be 18 nm through atomic force microscopy (XE-100, Park Systems, South Korea) in our previous study.[3] The wet thickness of the brushes in 0.1 mM and 1.0 mM NaCl solutions was characterized using another atomic force microscopy (BioScope Resolve, Bruker) and measured at 47.1 nm and 32.6 nm, respectively.[3]

Total internal reflection microscopy (TIRM)

As illustrated in the main text (Figure 1a), when a laser beam undergoes total internal reflection at the glass-water interface, evanescent waves form in the water near the interface and decay exponentially with increasing penetration distance. Due to gravity, PS microspheres in the water phase would get close to the glass-water interface and meet the evanescent waves. The scattering intensity from the PS microspheres follows a decay law:[4]

$$I(h) = I_0 e^{-\beta h}, \beta = \frac{4\pi}{\lambda}\sqrt{(n_1 sin\theta_i)^2 - {n_2}^2} \qquad (S1)$$

where n_1 and n_2 are the refractive indices of glass and water, respectively; θ_i is the angle of incidence; I_0 is the scattering intensity of microspheres stuck on the bottom surface; h is the distance between a tracked microsphere and a settled microsphere. By comparing the real-time scattering intensity with its maximum, the vertical location of a free-moving PS microsphere can be determined in real time.

For data analysis, the vertical Brownian motion of the microspheres near the surface is described by the Boltzmann distribution.

$$p(h) = Ae^{\frac{-\phi(h)}{k_B T}} \qquad (S2)$$

where $p(h)$ is the probability of finding a microsphere at a certain vertical location; A is a normalization constant; ϕ is the potential of mean forces; k_B is the Boltzmann constant; T is the temperature during the measurements. Then, by introducing a new parameter, h_m, to represent the location at which the microsphere appears with the highest frequency, the relative

interaction potential at different separation distances is then calculated from the vertical position distribution of the microsphere:

$$\frac{\phi(h)-\phi(h_m)}{k_BT}=ln\frac{n(h_m)}{n(h)} \tag{S3}$$

Then, according to the approach raised by Wu et al,[5] the ensemble-averaged profiles of interaction potential are integrated from individual ones to minimize the error caused by the size distribution of the PS microspheres. In typical TIRM measurements, the interaction potential profile is expected to obey a combination of net gravitational attraction and electrostatic repulsion:

$$\phi(h)=\phi_G(h)+\phi_E(h) \tag{S4}$$

The net gravitational attraction is simply given by

$$\phi_G(h)=Gh \tag{S5}$$

where G in this work is the net weight of the PS microsphere. By applying Derjaguin's approximation, the electrostatic repulsion can be described as[4]

$$\phi_E(h)=Be^{-\kappa h} \tag{S6}$$

with

$$B=16\varepsilon a\left(\frac{k_BT}{e}\right)^2\tanh\left(\frac{e\psi_1}{4k_BT}\right)\tanh\left(\frac{e\psi_2}{4k_BT}\right) \tag{S7}$$

where ε is the dielectric permittivity of water, a is the radius of the PS microsphere, e is the elemental charge, ψ_1 and ψ_2 are the Stern potentials of the microsphere and the plate, κ is the Debye diameter, respectively.

As Eqn. S4 has a minimum at

$$\kappa h_m=ln\frac{\kappa B}{G} \tag{S8}$$

B can be eliminated between Eqn. S4 and Eqn. S8 to obtain the relative interaction potential:

$$\frac{\phi(h)-\phi(h_m)}{k_BT}=\frac{G}{\kappa k_BT}\left(e^{-\kappa(h-h_m)}-1\right)+\frac{G}{k_BT}(h-h_m) \tag{S9}$$

Sometimes van der Waals attraction is also included in the range of measurements. In these cases, Eqn. S4 becomes

$$\phi(h)=\phi_G(h)+\phi_E(h)+\phi_{vdW}(h) \tag{S10}$$

Theoretically, the attraction part can be described by the Lifshitz–Hamaker equation:[6]

$$\phi_{vdW}(h)=-\frac{A(h)}{6}\left(\frac{2a}{h}\frac{h+a}{h+2a}-ln\frac{h+2a}{h}\right) \tag{S11}$$

where $A(h)$ is the retarded Hamaker constant of the interaction. However, the fitting of van der Waals attraction is challenging due to factors such as surface roughness and the retardation of the Hamaker constant. Previous TIRM studies have reported that Dejaguin's approximation and the Lifshitz-Hamaker equation (Eqn. S11) may not accurately describe the measured attraction.[6-8] In line with other researchers, an empirical exponential function was employed in this study to achieve a better fit for the attraction.[6,9]

Experimental setup of total internal reflection microscopy (TIRM)

In the experimental setup, a PCBMA/POEGMA-grafted glass slide and a bare glass slide were separated by a silicone gasket to assemble a sandwich-like cell. Two needles were used to penetrate the cell through the gasket for solution exchange. Sulfate PS microspheres were diluted to 0.8 mg/L with various concentrations of NaCl solution to act as tracers in TIRM measurements and serve as a model for contaminants in the system. Interparticle interactions are negligible at this tracer concentration since the average particle-particle distance is large in the sample cell, allowing for reliable measurements of particle-surface interactions. A 70° prism (BK-7, Shanghai Optics, USA) was attached to the bottom to correct the angle of incidence for total internal reflection. Any gap between the prism and the glass slide was filled with immersion oil (Type F, n = 1.5180, Leica, Germany). A He-Ne laser beam (λ = 632.8 nm, 35 mW, Research Electro-Optics, USA) was totally reflected at the glass-water interface to generate the evanescent waves. The scattered signal from the tracers was captured using an optical microscope (Olympus, Japan) equipped with a high-speed sCMOS camera (Zyla, Andor, UK). During the measurements, in order to further ensure the results were not influenced by interparticle interactions, we monitored the Brownian motion of PS microspheres under the microscope to avoid areas where the microspheres might aggregate. 100,000 images (512 × 512, 16 bits) were taken at a rate of 418 frames per second to track a single PS microsphere. The zero point of the separation distance discussed later was determined by the scattering intensity of tracers stuck to the bottom glass slide in a 100 mM NaCl solution. Image processing and data analysis were performed using custom MATLAB (MathWorks, USA) codes.

References

(1) Li, W.; Cao, F.; He, C.; Ohno, K.; Ngai, T. Measuring the In-teractions between Protein-Coated Microspheres and Polymer Brushes in Aqueous Solutions. Langmuir 2018, 34 (30), 8798-8806.

(2) Li, L.; Nakaji-Hirabayashi, T.; Kitano, H.; Ohno, K.; Kishioka, T.; Usui, Y. Gradation of proteins and cells attached to the surface of bio-inert zwitterionic polymer brush. Colloids and Surfaces B: Biointerfaces 2016, 144, 180-187.

(3) Wu, J.; Cao, F.; Yeung, P. W. F.; Li, M.; Ohno, K.; Ngai, T. A Total Internal Reflection Microscopy (TIRM)-Based Approach for Direct Characterization of Polymer Brush Conformational Change in Aqueous Solution. ACS Macro Letters 2024, 13 (10), 1376-1382.

(4) Prieve, D. C. Measurement of colloidal forces with TIRM. Advances in Colloid and Interface Science 1999, 82 (1-3), 93-125.

(5) Wu, H.-J.; Bevan, M. A. Direct measurement of single and ensemble average particle−surface potential energy profiles. Langmuir 2005, 21 (4), 1244-1254.

(6) Bevan, M. A.; Prieve, D. C. Direct measurement of retarded van der Waals attraction. Langmuir 1999, 15 (23), 7925-7936.

(7) Nayeri, M.; Abbas, Z.; Bergenholtz, J. Measurements of screening length in salt solutions by total internal reflection microscopy: Influence of van der Waals forces and instrumental noise. Colloids and Surfaces A: Physicochemical and Engineering Aspects 2013, 429, 74-81.

(8) Dagastine, R. R.; Bevan, M.; White, L. R.; Prieve, D. C. Calculation of van der Waals forces with diffuse coat-ings: applications to roughness and adsorbed polymers. The Jour-nal of Adhesion 2004, 80 (5), 365-394.

(9) Bevan, M. A.; Prieve, D. C. Forces and hydrodynamic in-teractions between polystyrene surfaces with adsorbed PEO− PPO− PEO. Langmuir 2000, 16 (24), 9274-9281.
